# Transcriptional profiles of peripheral eosinophils in chronic obstructive pulmonary disease and asthma—An exploratory study

**DOI:** 10.1111/jcmm.70110

**Published:** 2024-10-18

**Authors:** Katarzyna Mycroft, Małgorzata Proboszcz, Magdalena Paplińska‐Goryca, Rafał Krenke, Katarzyna Górska

**Affiliations:** ^1^ Department of Internal Medicine, Pulmonary Diseases and Allergy Medical University of Warsaw Warsaw Poland

**Keywords:** asthma, blood, COPD, eosinophil, IL‐13, RNA‐Seq, transcriptomic analysis

## Abstract

The role of eosinophilic inflammation in the pathogenesis of chronic obstructive pulmonary disease (COPD) remains ambiguous and likely differs from its role in asthma. The molecular processes underlying the differences between eosinophils from asthma and COPD have not been sufficiently studied. The objective of this study was to compare the transcriptomic profiles of blood eosinophils in COPD and asthma. Eosinophils were isolated from peripheral blood drawn from stable mild‐to‐moderate COPD and asthma patients. RNA was isolated from eosinophils and sequenced using an NGSelect RNA. The prepared libraries were sequenced on an Illumina platform. The study group included five patients with asthma and four patients with COPD. The RNA‐Seq data analysis identified 26 differentially expressed genes between COPD and asthma (according to adjusted *p*‐value). In total, 6 genes were upregulated (e.g. *CCL3L1*, *CCL4L2*, *GPR82*) and 20 were downregulated (e.g. *JUN*, *IFITM3*, *DUSP1*, *GNG7*) in peripheral eosinophils of COPD patients compared to asthma. The genes associated with signalling of IL‐4 and IL‐13 pathways were downregulated in COPD eosinophils compared to asthma. In conclusion, blood eosinophils from COPD and asthma patients present different transcriptomic profiles suggesting their different function in pathobiology of both obstructive airway diseases. These differences might indicate the direction of the search of targeted therapy in COPD.

## 1 INTRODUCTION

Eosinophils have been traditionally attributed to asthma pathobiology, but growing evidence suggests their involvement in chronic obstructive pulmonary disease (COPD) as well.^1^ COPD and asthma differ in their pathogenesis and clinical course, but they share several clinical and biological features.^2^, ^3^ Elevated blood eosinophil counts in asthma and COPD patients are associated with better treatment response to inhaled corticosteroids.^4^ However, the efficacy of inhaled corticosteroids ICS in COPD is poorer than in asthma.^5^ Moreover, contrary to asthma,^6^ therapies targeting eosinophils (e.g. anti‐IL5(R)) have not been found effective in COPD.^7^

These differences in response to anti‐inflammatory treatment between COPD and asthma patients might be caused by the complexity of immune pathways in the airways.^8^ Recent work by our group revealed differences in eosinophil polarization between COPD and asthma patients.^9^ However, the molecular processes underlying these differences remain largely unexplored. In recent years, next‐generation sequencing (NGS) technologies have provided an in‐depth understanding of the eosinophil function at a transcriptional level in various tissues, allowing for comparison of gene expression profiles between different conditions.^10^ Since eosinophils constitute a low percentage of blood cells, analysis of their gene expression has been challenging. RNA sequencing (RNA‐seq) data analysis has revealed differences between the transcriptional profiles of eosinophils located in different tissues such as blood,^11^ adipose tissue,^11^ lung or bone marrow.^12^

It has been shown that circulating eosinophils from asthmatics vary from those of healthy controls in their gene expression profile and eosinophils from patients with different hypereosinophilic conditions exhibit similar gene expression profile.^13^ Yun et al. demonstrated that eosinophilic COPD and eosinophilic asthma share a similar blood transcriptomic profile with overlapping eosinophil‐specific gene expression.^14^ Conversely, George et al. reported that transcriptional profiles of bronchial brushes in eosinophilic COPD and asthma showed little overlap with *CST1* being the only gene associated with blood eosinophils count.^15^ These contradictory results could be explained by the use of different biological samples, such as blood versus epithelial brush. Alternatively, they might indicate the complexity of molecular mechanisms of inflammation in COPD and asthma.

The transcriptional profiles of isolated peripheral eosinophils of COPD and asthma patients have not yet been compared. Therefore, the present study was aimed to compare the mRNA expression profiles of blood eosinophils in COPD and asthma to minimize the interference from other cell types.

## 2 RESULTS AND DISCUSSION

### 2.1 Patients

Initially, 25 patients were recruited, but 16 samples were excluded due to low RNA concentration and unsuccessful library generation. The final study group included five patients with asthma and four patients with COPD. All subjects were White. Among the assessed variables, only smoking status was significantly different between these two groups (Table S1). We found that among patients with inadequate RNA quality the majority (62.5%) were current smokers, whereas among patients with adequate RNA quality, current smokers constituted only 11%. This suggests that current smoking might have impacted eosinophil RNA quality and integrity. It is well known that smoking significantly impacts pattern of mRNA expression and induces cytotoxic, mutagenic and oxidative stress effects on cells.^16^ Cigarette smoke contains over 7000 chemicals, including well‐characterized ones such as acrolein, formaldehyde, acetaldehyde, which could impair RNA stability and integrity.^16^ Data on RNA quality, concentration and eosinophil numbers in each subject are presented in Table S2. The subjects' characteristics are presented in Table 1. At the time of the study, none of the COPD patients were receiving ICS, three of the five asthma patients were on low‐dose ICS, and none of the studied patients received biological treatment or oral steroids. The COPD patients were all but one ex‐smokers, the median smoking load of 40 (IQR 30–80) pack‐years (Table 1).

**TABLE 1:** Subject characteristics.

	COPD (*n* = 4)	Asthma (*n* = 5)	*p*
Age (years)	68.5 (59–73)	66 (47–72)	0.62
Gender (f/m)	1/3	3/2	0.36
BMI (kg/m^2^)	29.5 (27.2–35)	32.5 (28.4–32.7)	0.90
FEV_1_ (L)	2.0 (1.77–2.5)	2.7 (2.4–3.5)	0.39
FEV_1_ (% pred.)	69.5 (64–76.5)	88 (84–99)	0.027
Packyears	40 (30–80)	0	0.01
Atopy, *n*	0	2	0.22
Eosinophils, *n*	237.6 (129–354)	160 (141–311)	0.71
Eosinophils, %	3.5 (1.5–5.0)	2 (1.5–5.1)	0.80

*Note*: Data are presented as median (interquartile range) or number.

### 2.2 RNA‐seq analysis

The RNA‐Seq data analysis identified 26 differentially expressed genes (DEGs) between COPD and asthma (according to adjusted *p*‐value). In total, 6 genes were upregulated and 20 were downregulated in eosinophils of COPD patients compared to asthma (Table 2).

**TABLE 2:** Genes significantly up‐ or downregulated in blood eosinophils of patients with COPD compared to asthma. Genes downregulated in COPD are at the same time upregulated in asthma and vice versa.

	Gene symbol	Gene name	Protein function	Fold change	Adjusted *p*‐value
Genes significantly upregulated in COPD	*CCL3L1*	C‐C motif chemokine ligand 3 like 1	Chemokine	2.40	0.0048
	*CCL4L2*	C‐C motif chemokine ligand 4 like 2	Chemokine	2.35	0.0555
	*RSAD2*	Radical S‐adenosyl methionine domain containing 2	Antiviral response and innate immune signalling	2.23	0.0250
	*SERPINB2*	Plasminogen activator inhibitor 2	Protease inhibitor	2.21	0.0763
	*PRSS21*	Serine protease 21	Serine protease	2.21	0.0636
	*GPR82*	G protein‐coupled receptor 82	G‐protein coupled receptor	2.01	0.0930
Genes significantly upregulated in asthma	*SCARF1*	Scavenger receptor class F member 1	Membrane traffic protein	−2.02	0.0620
	*ENSG00000263244*			−2.03	0.0555
	*ZNF467*	Zinc finger protein 467	C2H2 zinc finger transcription factor	−2.04	0.0816
	*ENSG00000272916*		IncRNA	−2.04	0.0556
	*LINC02035*	Long intergenic non‐protein coding RNA 2035	IncRNA	−2.07	0.0717
	*NOL4L*	Nucleolar protein 4 like	Nuclear protein, RNA binding	−2.16	0.0897
	*JUNB*	Transcription factor jun‐B	Basic leucine zipper transcription factor	−2.17	0.0477
	*BCL6*	B‐cell lymphoma 6 protein	C2H2 zinc finger transcription factor	−2.17	0.0897
	*PELATON*	Plaque enriched LncRNA In atherosclerotic and inflammatory bowel macrophage regulation	IncRNA	−2.20	0.0625
	*ENSG00000268903*		Pseudogene	−2.22	0.0897
	*CNTNAP3*	Contactin‐associated protein‐like 3	Cell adhesion molecule	−2.22	0.0897
	*LINC00963*		IncRNA	−2.30	0.0636
	*GNG7*	Guanine nucleotide‐binding protein G(I)/G(S)/G(O) subunit gamma‐7	Heterotrimeric G‐protein	−2.34	0.0477
	*ENSG00000280138*		Uncategorized gene	−2.34	0.0048
	*ENSG00000228327*	General transcription factor IIi (GTF2I) pseudogene	Pseudogene	−2.35	0.0556
	*SLC25A37*	Mitoferrin‐1	Mitochondrial iron transporter	−2.40	0.0556
	*ZNF107*	Zinc finger protein 107	C2H2 zinc finger transcription factor	−2.41	0.0556
	*DUSP1*	Dual specificity protein phosphatase 1	Protein phosphatase	−2.42	0.0477
	*IFITM3*	Interferon induced transmembrane protein 3	Defence/immunity protein	−3.26	0.0015
	*JUN*	Transcription factor AP‐1	Basic leucine zipper transcription factor	−3.30	0.0015

Abbreviation: LncRNA, long non‐coding RNA.

**FIGURE 1:**
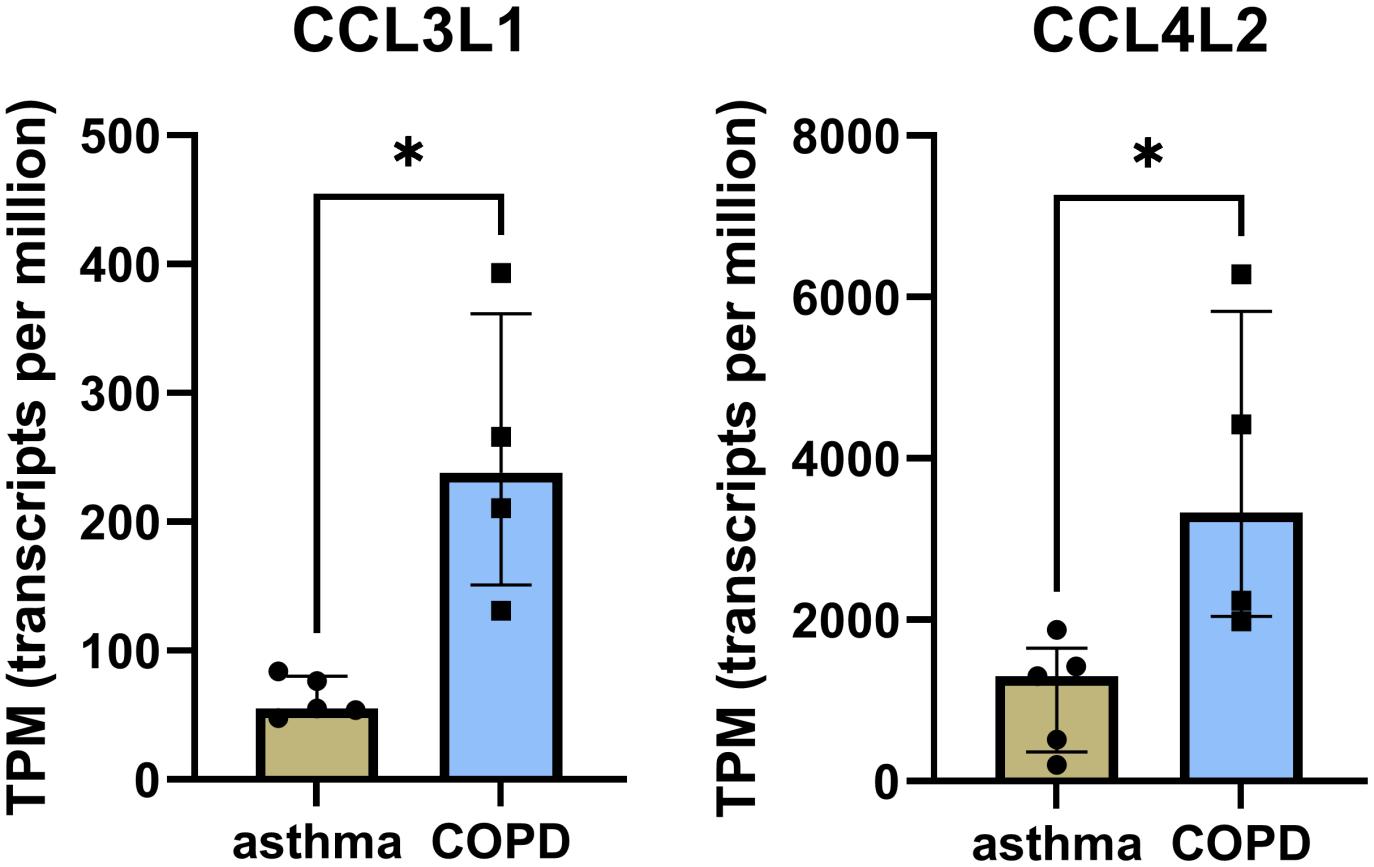
Dot plots showing the expression of *CCL3L1* and *CCL4L2* in COPD and asthma patients.

The most strongly upregulated genes in COPD (2.40‐fold change and 2.35‐fold change, respectively) were *CCL3L1* and *CCL4L2*, which encode chemokines. Dot plots showing the transcripts per million (TPM) values of *CCL3L1* and *CCL4L2* in COPD and asthma patients are presented in Figure 1. *CCL3L1* and *CCL4L2* expression is largely driven by copy number variation which is dependent on race.^17^ However, in the present study we found that all COPD subjects had increased expression levels of both genes compared to asthma. Interestingly, we found that *CCL3L1*, but not *CCL3*, was upregulated in eosinophils of COPD patients. Both *CCL3L1* and *CCL3* are isoforms of macrophage inflammatory protein 1α (MIP‐1α), with the former exhibiting a sixfold greater affinity to the receptor CCR5.^17^ MIP‐1α‐CCR5 binding is linked to the processes associated with tight junction injury in the airway epithelium in COPD.^18^ CCR5 also serves as a receptor for MIP‐1β encoded by i.a. CCL4L2 which in our study was found to be upregulated in COPD patients. Both MIP‐1α and MIP‐1β are monocyte and macrophage chemoattractants. It has been shown that MIP‐1α plays a negligible role as an eosinophil chemoattractant,^19^ unlike MIP‐1β.^20^ Increased expression of MIP‐1α in COPD patients has been linked to cigarette smoke exposure.^18^ However, the MIP‐1β isoform encoded by *CCL4L2* has an unknown affinity to CCR5 and its biological function has not been studied.^21^ The upgraded mRNA expression of *CCL3L1* in COPD eosinophils found in our study may suggest that in COPD eosinophils attract macrophages into the lungs rather than drive local eosinophilic inflammation. This finding underscores the different function of eosinophils in asthma and COPD pathobiology and might explain different ICS‐sensitivity of patients with COPD and asthma. Keeping in mind a small sample size in this analysis, these results should be interpreted carefully and confirmed in larger cohorts.

Other genes upregulated in COPD eosinophils in our study include protease inhibitor (*SERPINB2*), serine protease (*PRSS21*) and G‐protein coupled receptor (*GPR82*). Of these, *GPR82* has previously been identified as an eosinophil signature gene,^22^ but in fact, its role in the pathogenesis of eosinophilic inflammation remains unknown. *SERPINB2* upregulation in COPD patients found in our study is surprising. *SERPINB2* is typically induced by IL‐13 and elevated in eosinophilic asthma patients.^23^, ^24^ In COPD however, the *SERPINB2* expression was found not to be different between eosinophilic and non‐eosinophilic COPD patients.^25^ Although *SERPINB2* is known to be downregulated with corticosteroid treatment,^24^ additional analysis did not reveal significant differences in expression level between asthmatics receiving ICS and those without ICS (*p* = 0.39). However, these results must be interpreted with caution due to the low sample size included in our study.

The two most strongly upregulated genes (over 3‐fold) in asthmatic eosinophils revealed in our analysis, *JUN* and *IFITM3*, have already been reported to be involved in asthma pathogenesis. *IFITM3* belongs to the *IFITM* family which encodes homologous proteins localized in the plasma and endolysosomal membranes. It has been shown that IFITM deficiency reduces allergic inflammation in a murine model of asthma.^26^ Moreover, increased expression of *JUN* in PBMC, the transcription factor known to upregulate other inflammatory genes has been previously observed in patients with severe asthma compared to mild‐to‐moderate asthma or healthy controls.^27^ The protein encoded by *JUN* is involved in MAPK signalling cascades^28^ and might contribute to severe asthma pathogenesis by causing corticosteroid resistance.^29^ Interestingly, we found upregulation of *DUSP1* in asthma patients which might suggest an expectation of downregulated JUN expression, as DUSP1 attenuates MAPK signalling cascade. Our study implies that MAPK signalling in eosinophils is complex and dependent on many signal inducers. Moreover, *DUSP1* upregulation is known to be induced by ICS treatment.^28^ Although *DUSP1* expression trended higher in ICS‐users (*n* = 3) compared to ICS‐non‐users (*n* = 6), it did not achieve statistical significance (*p* = 0.09), probably due to the small sample size. Other genes downregulated in eosinophils of COPD compared to asthma patients in our study encoded membrane proteins (*GNG7, CNTNAP3, SCARF1*) and transcription factors (*ZNF107, BCL6, JUNB, ZNF467*). A volcano plot was used to visually present the detailed analysis of differentially expressed genes between blood eosinophils from COPD and asthma patients (Figure S1).

### 2.3 Enrichment and pathway analyses

**FIGURE 2:**
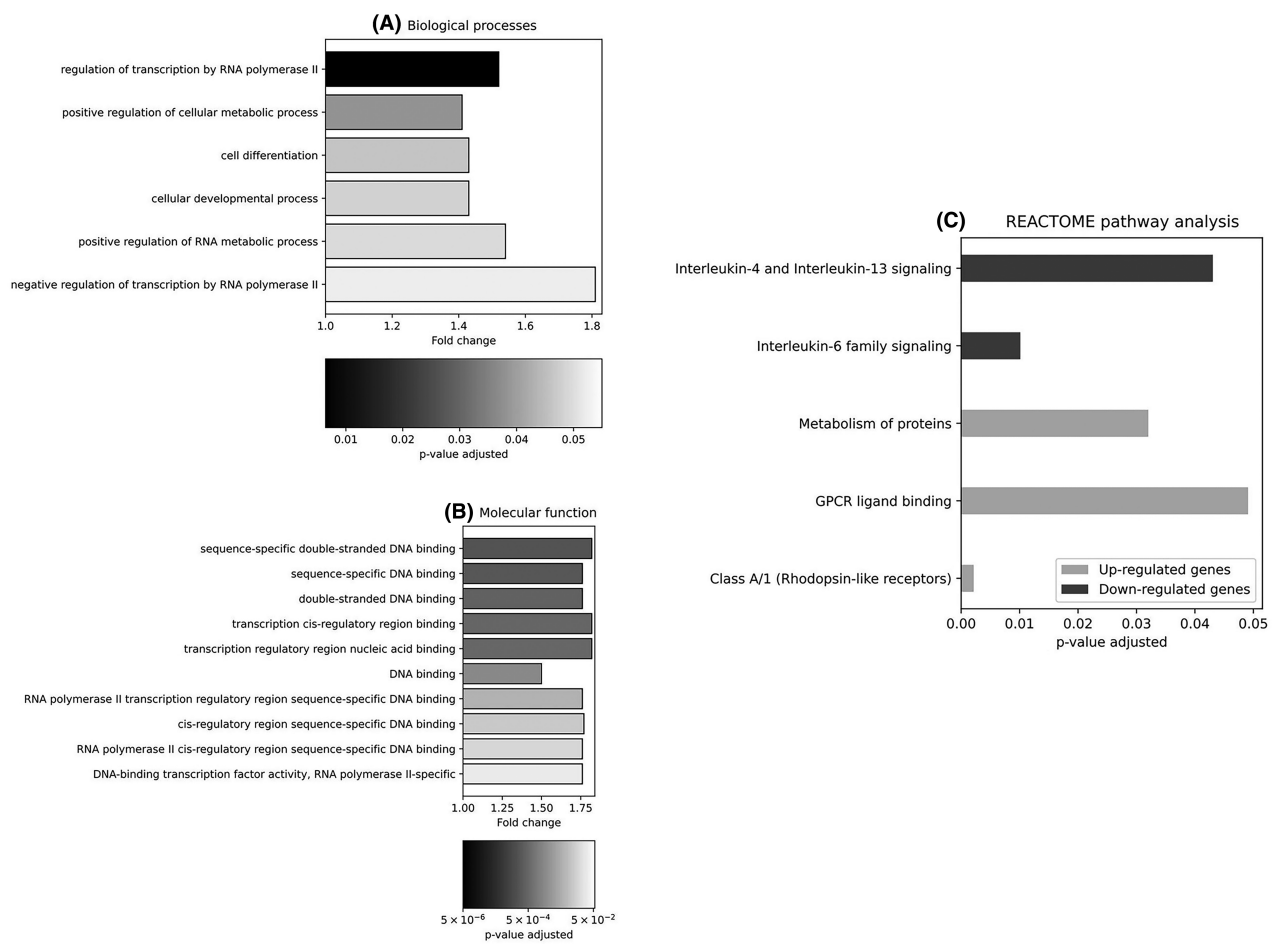
(A, B) Gene ontology analysis of the downregulated genes selected among 10% of genes with the lowest *p*‐value. The bar chart showing the GO terms for biological processes (A) and molecular function. (B) The *x*‐axis represents the fold change. (C) The REACTOME significantly enriched pathways among the top 10% of genes (up‐ and downregulated analysed separately). Analysis according to the adjusted *p*‐value.

The Gene Ontology (GO) enrichment analysis of the downregulated genes in COPD versus asthma eosinophils revealed association with negative regulation of transcription by RNA polymerase II (GO:0000122), positive regulation of RNA metabolic process (GO:0051254) and cell differentiation (GO:0030154) (Figure 2A). The molecular function terms of the downregulated genes in COPD versus asthma eosinophils were associated with DNA binding (GO:0003677), including: DNA‐binding transcription factor activity, RNA polymerase II‐specific (GO:0000981), RNA polymerase II cis‐regulatory region sequence‐specific DNA binding (GO:0000978) and RNA polymerase II transcription regulatory region sequence‐specific DNA binding (GO:0000977) (Figure 2B). Kyoto Encyclopedia of Genes and Genomes (KEGG) pathway analysis did not reveal any significantly overrepresented pathways. However, REACTOME pathway analysis indicated five overrepresented pathways (three for the upregulated and two for the downregulated genes) in COPD compared to asthma eosinophils (Figure 2C). Significant associations were found with pathways involving Rhodopsin‐like receptors, G protein‐coupled receptors (GPCR) ligand binding and metabolism of proteins (for the upregulated genes) and signalling of IL‐6 family, IL‐4 and IL‐13 (for the downregulated genes) in COPD eosinophils. IL‐4 and IL‐13, alongside IL‐5 are the key cytokines in type 2 (Th2) inflammation. Our results confirm that Th2 inflammation is an important pathway in asthma pathophysiology but to a lesser extent in COPD. Similar conclusions were made by other authors suggesting that the differences in the transcriptional profiles in COPD and asthma patients might reflect different mechanisms driving eosinophilic inflammation in COPD.^20^ On the other hand, the promising results of a recent dupilumab trial among severe eosinophilic COPD patients^30^ suggests greater involvement of IL‐4/IL‐13 genes and therefore of the Th2 pathway in COPD pathobiology.

## 3 LIMITATIONS

The analysis of transcriptomic profiles of peripheral eosinophils in humans is challenging due to their low number in the circulation and the difficulty to obtain high‐quality RNA as reported by other authors.^13^ Even though elevated blood eosinophil levels ≥300 cells/μL are found in up to 24% of COPD, significant eosinophilia is rare in COPD. Moreover, eosinophil levels can vary throughout the day and between stable and exacerbated state of the disease.^1^ Some authors overcame these difficulties by analysing the whole blood and identifying eosinophilic gene signatures.^14^ In our study, we attempted the single cell eosinophilic analysis as a more precise and detailed evaluation of eosinophil function in COPD versus asthma. However, we did not assess the associations between eosinophil gene expression and the microenvironment (e.g. cytokine levels) which could have impacted the transcriptional profiles of eosinophils. Eosinophil gene expression is also dependent on the activation status which was not evaluated in the present study. Moreover, corticosteroid treatment profoundly impacts has a huge impact on the gene expression; it is estimated that between 1000 and 2000 genes are regulated by the glucocorticoid receptor.^31^ Patients included to our study were treated with low‐dose ICS, and it is known that only a small fraction of them enters the bloodstream. On the other hand, patients on ICS treatment are more symptomatic and have a higher level of inflammatory mediators. Bearing in mind the possible impact of ICS on mRNA expression further evaluation of the results in untreated patients is needed. Finally, we are aware that the results of the present study are hampered by the small sample size. Considering the exploratory character of this study, the evaluation of these results in larger group and verification with other methods (PCR) will be needed in the future.

## 4 CONCLUSIONS

Peripheral eosinophils from COPD and asthma patients exhibit distinct transcriptomic profiles suggesting their different function in pathobiology of both obstructive airway diseases.

In COPD, eosinophils show upregulation of CCL3L1 and CCL4L2, potentially promoting macrophage‐mediated inflammation with less involvement in Th2 inflammation. These differences might indicate the direction of the search of targeted therapy in COPD.

## AUTHOR CONTRIBUTIONS

**Katarzyna Mycroft:** Conceptualization (equal); data curation (equal); formal analysis (equal); investigation (lead); methodology (supporting); visualization (lead); writing – original draft (lead). **Małgorzata Proboszcz:** Investigation (equal); methodology (equal); writing – review and editing (equal). **Magdalena Paplińska‐Goryca:** Conceptualization (equal); formal analysis (equal); investigation (equal); methodology (equal); supervision (equal); writing – review and editing (equal). **Rafał Krenke:** Supervision (equal); validation (equal); writing – review and editing (equal). **Katarzyna Górska:** Conceptualization (equal); formal analysis (equal); supervision (equal); writing – review and editing (equal).

## FUNDING INFORMATION

This research was partly funded by the National Science Centre, Poland (UMO‐2019/35/B/NZ5/00694).

## CONFLICT OF INTEREST STATEMENT

The authors declare no conflicts of interest.

## Supporting information


Data S1.

## Data Availability

The RNA‐Seq data were uploaded to GEO Omnibus (reference no. GSE237284).
