# Supplementary material for: Transcriptional profiles of peripheral eosinophils in chronic obstructive pulmonary disease and asthma—An exploratory study
Source: J Cell Mol Med. 2024 Oct 18;28(20):e70110. doi: 10.1111/jcmm.70110 (PMC11487681; doi:10.1111/jcmm.70110)
Supplement: Supplementary file 1 — Data S1. [file JCMM-28-e70110-s001.docx]

**Methods**

*Patients*

Patients with stable, mild-to-moderate COPD and asthma, having blood eosinophil counts ≥100 cells/µL in the year preceding the study, scheduled for the outpatient visit in the Department of Internal Medicine, Pulmonary Diseases and Allergy of the Medical University of Warsaw between October 2020 and February 2022 were enrolled in the study. The threshold of 100 cells/µL for blood eosinophils was based on the results of clinical trials which assessed the responsiveness of COPD patients to ICS and what has been also reflected in GOLD recommendations [1].

Inclusion criteria for COPD group were as follows: 1/ the age of 40 years and over, 2/ diagnosis of COPD based on GOLD 2020 recommendations, i.e. the past medical history, history of smoking ≥10 packyears, typical signs and symptoms [1] and irreversible airway obstruction found in spirometry (post-bronchodilator FEV1/FVC below the lower limit of normal [2] 3/ blood eosinophils ≥100 cells/µL proven at least once in the year preceding the study.

Inclusion criteria for asthma were as follows: 1/ the age of 18 years and over, 2/ diagnosis of asthma based on GINA 2020 recommendations, i.e. past medical history, typical signs and symptoms and demonstration of variable expiratory airflow limitation [3], 3/ a negative smoking history, 4/ blood eosinophils ≥100 cells/µL demonstrated at least once in the year preceding the study.

The exclusion criteria were: 1/ a concomitant COPD and asthma diagnosis; 2/ COPD or asthma exacerbation requiring treatment with systemic corticosteroid and/or antibiotics within the previous 3 months; 3/ conditions that could have affected eosinophil count and function: other chronic or acute lung disease, autoimmune and haematological diseases, malignancies, severe cardiovascular diseases; 4/ biologicals, systemic corticosteroids, or other immunosuppressive treatment.

Clinical and demographic data (including exacerbation history, comorbidities, medications) were collected on the visit. COPD patients performed COPD Assessment Test (CAT) and modified Medical Research Council (mMRC) dyspnea scoring, while asthma patients completed the Asthma Control Test (ACT). Spirometry was performed according to the ATS/ERS guidelines [4]. Atopy was diagnosed when either at least one skin prick test for inhalant allergens was positive (a mean wheal diameter ≥3 mm) or specific IgE level for inhalant allergens was over 0.35 kU/L.

*Blood sampling and processing*

Venous blood (15 ml collected in EDTA tube) was taken for peripheral eosinophil isolation in the morning (between 8 and 9 a.m.). Polymorphonuclear leukocytes were isolated by Lymphoprep™ (StemCell, Canada) centrifugation according to manufacturers’ protocol.

*Eosinophil isolation*

The EasySep™ Human Eosinophil Isolation Kit (StemCell, Canada) was used to isolate eosinophils from a polymorphonuclear cell-rich fraction of peripheral blood by negative selection. The isolated eosinophils were stored in RNAlater until RNA isolation.

*RNA isolation*

The RNA was isolated from eosinophils using column-based method with NucleoSpin RNA (Machery&Nagel, Germany) using the protocol including DNA digestion. The concentration and quality of the isolated RNA were determined using Nanodrop 2000 (Thermo Fisher Scientific, Waltham, MA, USA).

*RNA-seq Analysis*

After determining RNA integrity and quality, RNA was prepared and sequenced using an NGSelect RNA (NGS Built For You (eurofinsgenomics.eu)) product. This included purification of mRNA, fragmentation, strand-specific cDNA synthesis, end-repair, ligation of sequencing adapters, amplification, and purification. The prepared libraries were then quality-checked, pooled, and sequenced on an Illumina platform (Illumina NovaSeq6000, PE150 mode).

*Statistical Analysis*

Basic statistical analyses were performed using Statistica 13.3 software (StatSoft Inc., Tulsa, USA). Data are presented as median and interquartile range (IQR) or number and percentage. Differences between continuous variables were tested using nonparametric Mann–Whitney U test. Fisher’s exact test was used to test the differences between nominal variables.

Genes receiving less than 10 reads on an average across the compared groups were removed. The abundance counts of each gene were then used to perform differential gene expression (DGE). DGE was performed using R/Bioconductor DESeq2 package [5], which essentially normalizes the abundance counts to account for observed variance (due to differences in sequencing depths, sample groups and replicates) generating normalized gene counts. Statistical tests were performed for each gene to compare the distributions between conditions generating p-values for each gene. The final p-values were corrected by determining false discovery rates (FDR) using the Benjamin–Hochberg method. Using a FDR corrected p-value (adjusted p-value) <0.1 as a threshold and a fold change of 2 or more, significantly differentially expressed genes between conditions were identified and reported.

Additional analysis of the 10% up- and down-regulated genes (with the lowest p-value) was performed using PANTHER Overrepresentation Test (http://pantherdb.org/webservices/go/overrep.jsp, released 13 Oct 2022) and g:Profiler [6]. Gene ontology (GO) enrichment was calculated using Fisher’s Exact test with Bonferroni correction. The Kyoto Encyclopedia of Genes and Genomes (KEGG) categories and REACTOME pathway analysis were performed. The reference list used in the analysis contained all the genes detected in the experiment. Terms with a corrected p-value less than 0.05 were considered significantly enriched.

**References**

1. Global Strategy for the Diagnosis, Management and Prevention of COPD, Global Initiative for Chronic Obstructive Lung Disease (GOLD). 2020. Available online: http://goldcopd.org/ (accessed on 14 September 2020).
2. Quanjer, P.H.; Stanojevic, S.; Cole, T.J.; Baur, X.; Hall, G.L.; Culver, B.H.; Enright, P.L.; Hankinson, J.L.; Ip, M.S.M.; Zheng, J.; et al. Multi-Ethnic Reference Values for Spirometry for the 3–95-Yr Age Range: The Global Lung Function 2012 Equations. Eur. Respir. J. 2012, 40, 1324–1343.
3. GINA. Global Initiative for Asthma (GINA). Global Strategy for Asthma Management and Prevention. Global Initiative for Asthma—Global Initiative for Asthma—GINA. 2020. Available online: ginasthma.org (accessed on 14 September 2020).
4. Graham, B.L., Steenbruggen, I., Miller, M.R., Barjaktarevic, I.Z., Cooper, B.G., Hall, G.L., Hallstrand, T.S., Kaminsky, D.A., McCarthy, K., McCormack, M.C., et al. Standardization of Spirometry 2019 Update. An Official American Thoracic Society and European Respiratory Society Technical Statement. Am. J. Respir. Crit. Care Med. 2019, 200, e70–e88.
5. Love MI, Huber W, Anders S. Moderated estimation of fold change and dispersion for RNA-seq data with DESeq2. Genome Biol. 2014;15(12):550.
6. Raudvere U, Kolberg L, Kuzmin I, Arak T, Adler P, Peterson H, et al. g:Profiler: a web server for functional enrichment analysis and conversions of gene lists (2019 update). Nucleic Acids Res. 2019;47(W1):W191-W8.

Supplementary Tables

Table S1. Comparison between subjects with adequate RNA quality (n=9) and inadequate RNA quality (n=16)

| Variable | Adequate RNA quality  N=9 | Inadequate RNA quality  N=16 | P value |
| --- | --- | --- | --- |
| Age (years) | 66 (53-72) | 63.5 (52-69) | 0.41 |
| Gender (f/m) | 4/5 | 7/9 | 0.65 |
| COPD/asthma | 4/5 | 10/6 | 0.32 |
| BMI | 31.5 (27.5-32.7) | 28.3 (26-31.8) | 0.20 |
| White blood cell count | 8.55 (6.49-9.68) | 7.35 (5.48-9.08) | 0.28 |
| Blood eosinophil count | 230 (140-260) | 195 (80-280) | 0.64 |
| Blood eosinophil percentage | 2 (1.5-5) | 3 (1.0-5) | 0.88 |
| Smoking status (never/current/ex) | 5/1/3 | 4/10/1 | 0.03 |
| Packyears | 40 (30-80) | 45 (30-51) | 0.86 |
| \| postFEV1 (L) \| \| --- \| | 2.37 (1.97-2.96) | 1.87 (1.64-2.61) | 0.65 |
| \| postFEV1 (%) \| \| --- \| | 80 (73-88) | 70.5 (61-87.4) | 0.47 |

Table S2. RNA quality, concentration and eosinophil numbers in the study group.

| **Sample number** | **diagnosis** | **quality analysis** | **Concentration (ng/µl)** | **A260** | **A280** | **260/280** | **eosinophil numbers (cells/ µl)*** |
| --- | --- | --- | --- | --- | --- | --- | --- |
| 1 | Asthma | No | 1.40 | 0.036 | 0.021 | 1.71 | 80 |
| 2 | COPD | No | 1.60 | 0.040 | 0.032 | 1.25 | 80 |
| 3 | COPD | No | 2.24 | 0.056 | 0.036 | 1.56 | 280 |
| 4 | COPD | No | 2.00 | 0.050 | 0.042 | 1.19 | 160 |
| 5 | asthma | yes | 1.80 | 0.045 | 0.032 | 1.41 | 140 |
| 6 | COPD | No | 1.84 | 0.046 | 0.028 | 1.64 | 60 |
| 7 | asthma | yes | 7.40 | 0.185 | 0.108 | 1.71 | 80 |
| 8 | COPD | No | 2.08 | 0.052 | 0.030 | 1.73 | 200 |
| 9 | COPD | yes | 6.52 | 0.163 | 0.089 | 1.83 | 180 |
| 10 | asthma | yes | 3.72 | 0.093 | 0.049 | 1.90 | 310 |
| 11 | COPD | yes | 6.84 | 0.171 | 0.084 | 2.04 | 260 |
| 12 | COPD | No | 4.44 | 0.111 | 0.067 | 1.66 | 260 |
| 13 | COPD | yes | 7.84 | 0.196 | 0.094 | 2.09 | 260 |
| 14 | asthma | No | 2.36 | 0.059 | 0.032 | 1.84 | 390 |
| 15 | COPD | yes | 8.16 | 0.204 | 0.101 | 2.02 | 230 |
| 16 | COPD | No | 2.88 | 0.0720 | 0.0350 | 2.06 | 80 |
| 17 | Asthma | No | 1.32 | 0.0330 | 0.0170 | 1.94 | 190 |
| 18 | asthma | yes | 3.12 | 0.0780 | 0.0170 | 4.59 | 480 |
| 19 | Asthma | No | 3.56 | 0.0890 | 0.0000 | ---- | 260 |
| 20 | Asthma | No | 2.96 | 0.074 | 0.046 | 1.61 | 220 |
| 21 | asthma | yes | 4.44 | 0.111 | 0.060 | 1.85 | 130 |
| 22 | Asthma | No | 0.20 | 0.005 | 0.002 | 2.50 | 840 |
| 23 | COPD | No | 0.20 | 0.005 | 0.001 | 5.00 | 330 |
| 24 | COPD | No | 0.16 | 0.004 | 0.002 | 2.00 | 120 |
| 25 | COPD | No | 0.24 | 0.006 | 0.004 | 1.50 | 260 |

Supplementary Figures

Figure S1

Volcano plot for the comparison between the blood eosinophils from COPD and asthma patients. The x-axis represents the log2 fold change (FC), while the y-axis indicates the log10 adjusted p-values. Red dots represent genes which were significantly up- or down-regulated with log2 FC≤1, green dots represent genes which were significantly up- or down-regulated with log2 FC>1, and black dots show non-significantly deregulated genes.
